# Changes in Bystander CPR Patterns of Private and Public Locations Before and After COVID-19: An Interrupted Time-Series of a Multicentre Out-of-Hospital Cardiac Arrest Cohort

**DOI:** 10.3390/jcm15145469

**Published:** 2026-07-13

**Authors:** Youdong Sohn, Gyuchong Cho, Youngsuk Cho, Taejin Park, the Korean Cardiac Arrest Research Consortium (KoCARC) Investigators

**Affiliations:** Department of Emergency Medicine, Kangdong Sacred Heart Hospital, Hallym University College of Medicine, Seoul 05355, Republic of Korea; medysohn@gmail.com (Y.S.); faith2love@kdh.or.kr (Y.C.); humcemergency@gmail.com (T.P.)

**Keywords:** out-of-hospital cardiac arrest, bystander cardiopulmonary resuscitation, COVID-19, interrupted time-series, rescue breathing, compression-only CPR

## Abstract

**Background**: The COVID-19 pandemic exerted competing pressures on bystander cardiopulmonary resuscitation (CPR)—infection control aversion to rescue breathing versus a shift in arrests toward the home—yet reported effects have been inconsistent and evidence by arrest location and CPR method is scarce. **Methods**: Using the Korean Cardiac Arrest Research Consortium multicentre registry (January 2016–June 2025; 21,182 adults with home or public out-of-hospital cardiac arrest), we performed an interrupted time-series analysis across pre-COVID-19, pandemic (February 2020–May 2023) and endemic periods. **Results**: Both settings shared rising pre-COVID-19 trends; at the February 2020 interruption, the bystander CPR rate trajectory changed asymmetrically (home −0.278 versus public −0.151 percentage points/month), with block-bootstrap counterfactual shortfalls by series end of −17.6 (home) and −12.5 (public) percentage points. After adjustment, the location interaction was marginal overall (*p* = 0.065) but significant in witnessed arrests (*p* = 0.046). Conventional CPR fell abruptly and uniformly (level-change odds ratio 0.22), while compression-only CPR rose above 97% with no recovery through the endemic period. Period-by-location interaction was non-significant for return of spontaneous circulation, survival and good neurological outcome. **Conclusions**: COVID-19 produced a robust, uniform shift in bystander CPR methods toward compression-only CPR and a more limited and suggestive location-asymmetric change in the bystander CPR rate (marginal overall, significant only in witnessed arrests), without differential effects on short-term outcomes.

## 1. Introduction

Out-of-hospital cardiac arrest (OHCA) remains a leading cause of death worldwide and early bystander cardiopulmonary resuscitation (CPR) is critical to survival [1,2]. By early 2020, Korea had achieved sustained year-on-year increases in bystander CPR alongside a national public-access defibrillation programme [3,4], consistent with international resuscitation council emphasis on rate improvement and compression-only CPR [5,6].

The COVID-19 pandemic introduced two competing pressures: infection control concerns made mouth-to-mouth rescue breathing aversive, with interim councils recommending compression-only CPR for untrained or exposed bystanders [7,8], and stay-at-home behaviour shifted OHCA toward residential settings, where bystanders are usually family and public-access AEDs unavailable [9,10]. These pressures predict a method shift toward compression-only and a location-differential change in bystander CPR rate.

In Korea, the pandemic response unfolded in phases: a national infectious disease alert escalated in late January 2020 to its highest level the following month, graded social distancing measures were tightened and eased through 2020–2022, and stepwise normalisation led to the 2023 endemic transition [11]. These phases plausibly exerted time-varying effects on both the willingness to perform bystander CPR and the choice of CPR method. Existing evidence, however, is largely cross-sectional or confined to acute pandemic months, reports aggregate bystander CPR rates without distinguishing residential from public arrests, and seldom addresses the method of bystander CPR or applies a formal trend break framework [9,10,12,13,14,15,16,17]. We therefore analysed a multicentre Korean OHCA registry (2016–2025) using interrupted time-series (ITS) methodology [18,19] to track these changes across the pre-COVID-19, pandemic and endemic phases—characterising bystander CPR rate and method by private (home) versus public location, separating immediate level change from secular trend, and testing the location-asymmetric rate and method-uniform shift hypotheses. This study adds three elements that previous reports have not combined: (i) a formal interrupted time-series spanning the full pre-pandemic, pandemic and endemic phases (2016–2025) rather than a single acute-pandemic window; (ii) separation of home (private residence) from public arrests; and (iii) decomposition of the method of bystander CPR (compression-only versus conventional), not only its rate.

## 2. Materials and Methods

### 2.1. Study Design and Data Source

We performed a retrospective analysis of prospectively collected data from the Korean Cardiac Arrest Research Consortium (KoCARC), a multicentre hospital-based research network that enrols adult (≥18 years) OHCA patients of presumed medical aetiology transported to 33 participating hospitals [20]. Standardised data are captured via a web-based case report form following the Utstein template (ClinicalTrials.gov NCT03222999). Cases registered between October 2015 and June 2025 were eligible; the partial 2015 records were excluded.

### 2.2. Period Stratification

Three periods were defined by month of arrest, aligned to Korea’s pandemic policy: pre-COVID-19 (January 2016–January 2020), pandemic (February 2020–May 2023) and endemic (June 2023–June 2025). Boundaries correspond to the 26 January 2020 Korea Centers for Disease Control and Prevention national alert (raised to its highest level in February 2020) and the May 2023 endemic transition (crisis level downgraded effective 1 June 2023 [11]), so the descriptive periods coincide with the interrupted time-series break points (Supplementary Table S1).

### 2.3. Location Classification

Arrest location was classified as home (private residence) versus public (industrial site, sports facility, road/outdoor, public building, educational facility); the term home (rather than private) is used for this stratum throughout. Nursing facilities (*n* = 1472) were analysed as a separate stratum because of staff-driven response and distinct end-of-life dynamics (Supplementary Table S6). Cases with an undefined arrest place (*n* = 3663) were excluded from the primary location-specific analysis and examined separately (Supplementary Tables S8 and S9).

### 2.4. Outcomes

The co-primary outcomes were as follows: (i) any bystander CPR (chest compressions with or without rescue breathing, among patients with classified bystander CPR status; monthly proportion); and (ii) conventional bystander CPR (chest compression plus rescue breathing as a proportion of bystander-performed CPR—method composition; quarterly proportion, given sparsity). Compression-only CPR (the complement of conventional CPR among performed cases) was reported as a mirror outcome. Secondary outcomes were bystander automated external defibrillator (AED) use, prehospital return of spontaneous circulation (ROSC), survival to hospital discharge and good neurological outcome (Cerebral Performance Category 1 or 2 at discharge).

### 2.5. Bystander CPR Method Classification

The KoCARC core variable for bystander CPR records three mutually exclusive categories: chest compression only; chest compression with rescue breathing (conventional); and not performed. This three-category coding was used directly; bystander AED use was a separate variable.

### 2.6. Statistical Analysis

Primary analyses were defined a priori in an internal analysis plan (not publicly pre-registered). Baseline characteristics were compared by Kruskal–Wallis (continuous) and chi-square (categorical) across periods. The primary inference was segmented regression Y(t) = β_0_ + β_1_·time + β_2_·post + β_3_·time-since-post + harmonics + ε(t), with post indicating the period from February 2020 onward [18,19]; β_2_ and β_3_ estimate the immediate level change and the slope change, respectively. Newey–West HAC standard errors were applied (maxlag from 4·(*n*/100)^(2/9), floor 3). The monthly proportions were modelled unweighted; as a sensitivity they were refitted weighted by the monthly number of arrests (weighted least squares), and residual diagnostics (Durbin–Watson, Ljung–Box and Cook’s distance) were examined. For the (sparse) conventional CPR outcome, we used individual-level segmented logistic regression with hospital-cluster robust SE, with the interruption coded at 2020Q1; because January 2020 falls in the pre-COVID-19 period, this coding was confirmed in two sensitivity analyses—a monthly model with a February 2020 break, and a quarterly model excluding the straddling 2020Q1 quarter. The any-CPR rate was additionally analysed at the individual level with location × post and location × time-since-post interaction terms (adjusted for age, sex, witnessed). Outcomes (ROSC, survival, good neurological outcome) were analysed by multivariable logistic regression with period × location interaction (joint LR test), adjusted for the Utstein covariates (the interaction was tested both without and with the potential mediators bystander CPR and bystander AED). Pre-specified sensitivity analyses included cut-point sensitivity (January/March/April 2020), a 3-segment ITS with a second knot at May 2023, placebo interruptions at January 2018 and January 2019, pre-period exclusions for the conventional CPR model, HAC lag and semi-annual harmonic sensitivity, witnessed-stratified ITS, and block-bootstrap CIs (B = 2000) for the counterfactual gap. Effect sizes are reported with 95% CIs; two-sided *p* < 0.05 indicated statistical significance. Analyses used Python 3.14.3 (pandas 2.3.3, statsmodels 0.14.6, scipy 1.17.1).

### 2.7. Ethics, Data and Code

The KoCARC registry and present analysis were approved by the Institutional Review Board of Hallym University Kangdong Sacred Heart Hospital (No. 2015-09-002-033), which waived informed consent. The study was conducted following the Declaration of Helsinki and reported following STROBE and Bernal–Cummings ITS guidance (Supplementary Checklist S1). KoCARC data are available on application to the consortium; the analytic Python code is available from the corresponding author on reasonable request. During the preparation of this manuscript, the authors used Claude Sonnet 4.5 (Anthropic, San Francisco, CA, USA) for the purposes of language editing, statistical code review and figure formatting. The authors have reviewed and edited the output and take full responsibility for the content of this publication.

## 3. Results

### 3.1. Cohort

**Figure 1:**
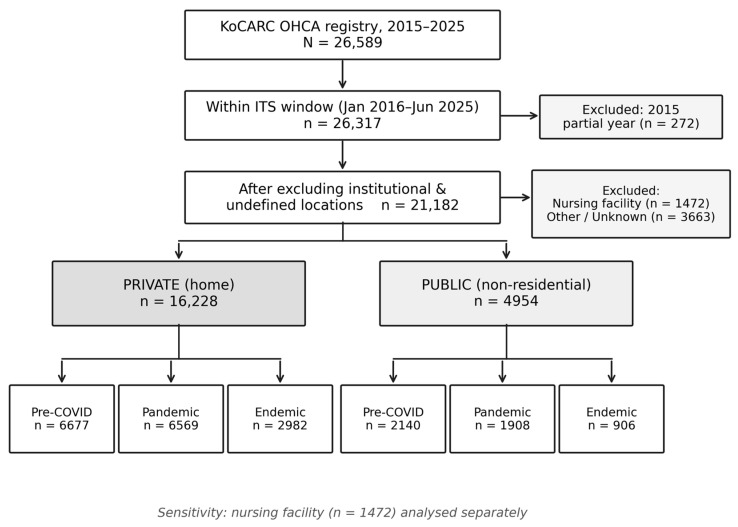
The study flow diagram. The flow of OHCA patients from the KoCARC registry (October 2015–June 2025) through the analytic cohort. After excluding 2015 partial-year cases, nursing facility arrests (analysed separately as a sensitivity stratum) and unclassified locations, 21,182 patients (private home 16,228; public 4954) formed the main cohort and were stratified into pre-COVID-19 (2016–January 2020), pandemic (February 2020–May 2023) and endemic (June 2023–2025) periods.

Of 26,589 cases in the KoCARC registry, 26,317 fell within the analytic window; after excluding nursing facility arrests (*n* = 1472, analysed separately) and undefined locations (*n* = 3663), 21,182 patients formed the main cohort (Figure 1; home 16,228, public 4954). Baseline characteristics by period are summarised in Table 1. The proportion of OHCA occurring at home rose from 75.7% (pre-COVID-19) to 77.5% (pandemic) and partly returned to 76.7% (endemic), consistent with stay-at-home behaviour during the acute pandemic (*p* = 0.023; Supplementary Figure S1). Age increased modestly across periods (65.8 to 68.5 years; *p* < 0.001), and witnessed-arrest proportion rose (57.9% to 61.7%; *p* < 0.001).

### 3.2. Bystander CPR Rate

**Figure 2:**
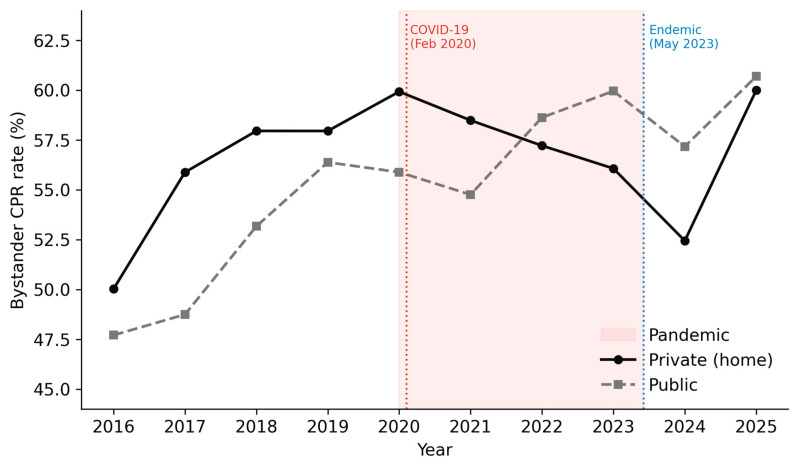
The annual bystander CPR rate by arrest location (2016–2025). Pink shading = pandemic period (February 2020–May 2023); red dotted line = interruption (February 2020); blue dotted line = endemic transition (May 2023). The private (home) rate peaked around 2020 and then declined, whereas the public rate continued to rise, producing a post-pandemic divergence and home/public crossover—the descriptive counterpart to Figure 3’s interrupted time-series.

**Figure 3:**
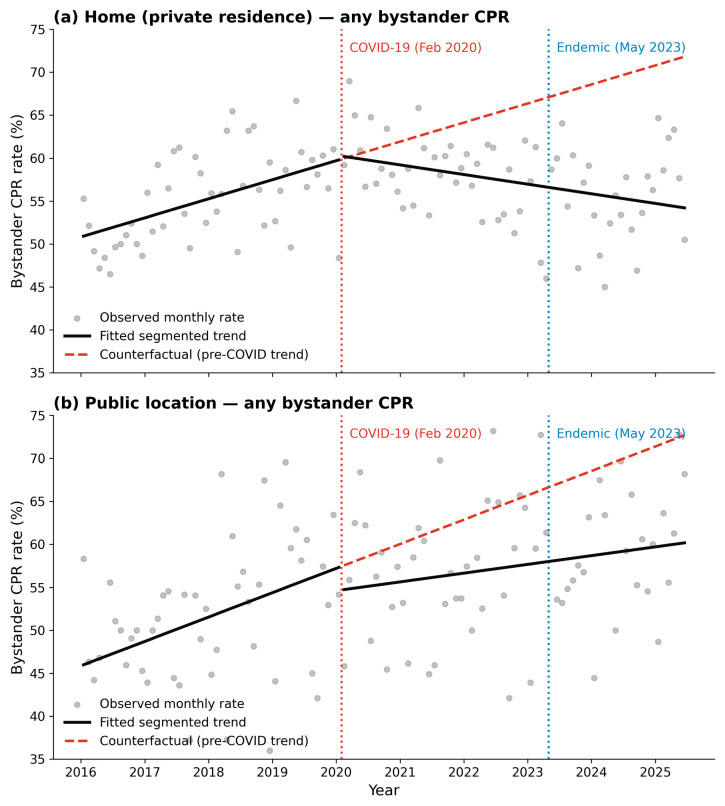
The interrupted time-series of any bystander CPR rate, monthly 2016–2025, by arrest location: (**a**) private (home); (**b**) public. Grey points = observed monthly proportions; solid black line = fitted segmented regression trend (Newey–West HAC standard errors, annual harmonics); red dashed line = counterfactual (pre-COVID-19 trend extrapolated); red dotted vertical line = interruption (February 2020). Both settings had similar rising pre-COVID-19 trends. After COVID-19, the slope changed significantly in both settings, but asymmetrically: at home, the trend reversed to a decline (post-COVID-19 net −0.093 pp/month); in public, the trend slowed but remained positive (+0.085 pp/month).

Crude rates were 54.5% (pre-COVID-19), 58.1% (pandemic) and 56.2% (endemic) overall (Table 1; Figure 2; Supplementary Figure S2). The ITS (Figure 3; Table 2) showed similar rising pre-COVID-19 trends at both locations (home +0.185 pp/month, public +0.236 pp/month; both *p* < 0.001), no statistically significant immediate level change at the COVID-19 interruption, and significant but asymmetric slope changes: home −0.278 pp/month (*p* < 0.001; post-COVID-19 net −0.093 pp/month) versus public −0.151 pp/month (*p* = 0.025; post-COVID-19 net +0.085 pp/month). Block-bootstrap 95% confidence intervals (CIs) for the cumulative gap versus counterfactual at series end were −17.6 pp (95% CI −26.5 to −9.1) at home and −12.5 pp (−23.1 to −2.7) in public; this counterfactual gap is the model-implied difference from the extrapolated pre-COVID-19 rising trend, not an absolute fall in the bystander CPR rate, which remained close to its pre-pandemic level (crude rates 54.5%, 58.1%, 56.2%). After individual-level adjustment for age, sex and witnessed status with hospital-cluster robust standard errors, the home slope change remained significant (*p* < 0.001) but the joint location × interruption interaction was only marginally significant (Wald W = 5.46, df = 2, *p* = 0.065; Supplementary Table S2). A three-segment specification with a second knot at May 2023 (Korea’s endemic declaration) indicated a non-significant attenuation of the home decline in the endemic period (pandemic slope −0.199, endemic slope +0.043 pp/month, slope change *p* = 0.14; Supplementary Table S3). A placebo interruption one year before COVID-19 detected a significant level break in the home rate series (January 2019; *p* < 0.001; Supplementary Table S3), indicating pre-COVID-19 structural instability specific to the home series, so the home slope change is interpreted as hypothesis-generating. Witnessed-stratified analysis showed that the location-asymmetric pattern was concentrated in witnessed arrests (joint interaction *p* = 0.046), whereas no significant asymmetry was seen in unwitnessed cases (*p* = 0.40; Supplementary Table S4); because these subgroup tests were not adjusted for multiplicity, the witnessed result is regarded as exploratory. The trend-change estimates were essentially unchanged when monthly proportions were weighted by the number of arrests, and residual diagnostics showed no material autocorrelation (Supplementary Table S10).

### 3.3. Bystander CPR Method

**Figure 4:**
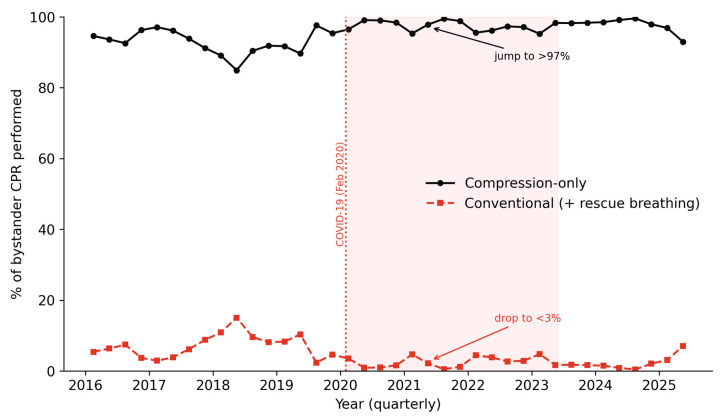
Bystander CPR method composition before and after COVID-19 (overall, quarterly). Compression-only CPR (solid black, circles) and conventional CPR with rescue breathing (dashed red, squares) as percentages of all bystander-performed CPR. Pink shading = pandemic period (February 2020–May 2023); red dotted line = interruption (February 2020). At the COVID-19 interruption, conventional CPR fell abruptly (level change OR 0.22, 95% CI 0.06–0.86; *p* = 0.030) and compression-only CPR rose correspondingly to over 97% of bystander-performed CPR with no recovery through 2025. The shift was near-identical across private (home) and public settings (see Table 3 and Supplementary Figures S6 and S7).

Across bystander-performed CPR (*n* = 11,490), conventional CPR (chest compression with rescue breathing) fell sharply from pre-COVID-19 to pandemic (6.8% → 2.5% at home; 9.0% → 2.6% in public; both *p* < 0.001) and did not recover (endemic 2.3% and 1.8%, respectively). The individual-level segmented logistic regression (Figure 4; Table 3) showed no significant pre-COVID-19 trend in conventional CPR (OR 1.034/quarter; *p* = 0.31), then an abrupt drop at the COVID-19 interruption (level change OR 0.22, 95% CI 0.06–0.86; *p* = 0.030), with no subsequent recovery (trend change *p* = 0.67). The shift was near-identical across settings (home OR 0.21 [0.05–0.86], *p* = 0.030; public OR 0.26 [0.07–0.96], *p* = 0.043); compression-only CPR became near-universal (>97%) through 2025. Considered a three-category composition of all OHCA with known status, the fall in conventional CPR was mirrored by a rise in compression-only CPR rather than by an increase in no bystander CPR, which did not rise during the pandemic (45.5% → 41.9% → 43.8%; Supplementary Figure S8). A pre-period sensitivity analysis showed that the level-change estimate was attenuated when 2018 was excluded (OR 0.30, *p* = 0.06), reflecting partial leverage by the 2018 conventional CPR peak; the direction of effect was consistent across all pre-period specifications (Supplementary Table S4). The abrupt level drop was reproduced when the interruption was re-coded as a February 2020 monthly break and when the straddling 2020Q1 quarter was excluded (Supplementary Table S11).

### 3.4. Bystander AED Use

Public-location bystander AED use was approximately eight-fold higher than at home (3.8–4.9% versus 0.5–0.6%) but did not change significantly across periods within either location (both *p* > 0.4; Table 1 and Supplementary Figure S3).

### 3.5. Outcomes

**Figure 5:**
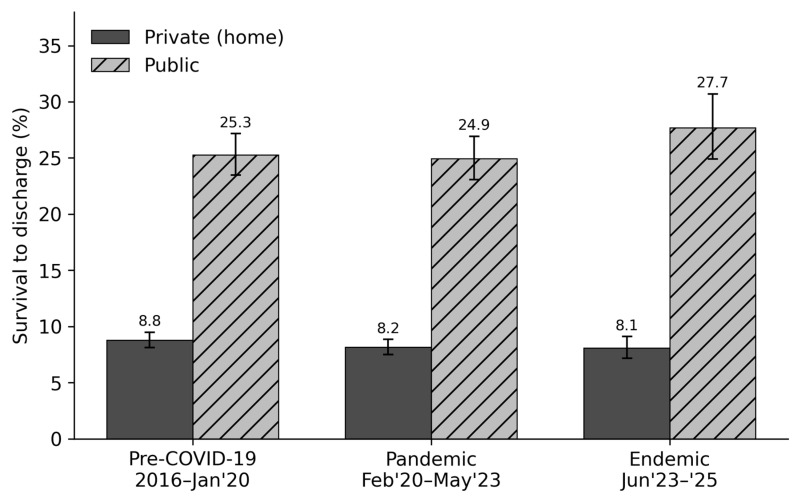
Survival to hospital discharge by arrest location and COVID-19 period. Wilson 95% confidence intervals are shown. The location paradox (approximately three-fold higher survival in public settings) was preserved across all periods; there was no pandemic collapse in either setting.

Prehospital return of spontaneous circulation (ROSC), survival to hospital discharge and good neurological outcome are summarised by location and period in Figure 5 and Table 1; the well-described location paradox (approximately three-fold higher rates in public versus home settings) was preserved across all periods. Multivariable logistic regression with period × location interaction (adjusted for age, sex, witnessed arrest, shockable rhythm, bystander CPR, bystander AED; hospital-cluster robust SE) showed no significant period × location interaction for any outcome (joint likelihood-ratio test: ROSC *p* = 0.18; survival *p* = 0.54; good neurological outcome *p* = 0.39; Supplementary Table S5), confirming that the pandemic did not differentially worsen short-term outcomes within either setting; removing the potential mediators (bystander CPR and bystander AED) from the model left the conclusion unchanged (ROSC *p* = 0.15; survival *p* = 0.32; good neurological outcome *p* = 0.34; Supplementary Table S12). Sensitivity analyses, including the nursing facility stratum and cut-point/placebo robustness checks, are reported in the Supplementary Material.

## 4. Discussion

In this multicentre Korean OHCA cohort, the COVID-19 interruption left a two-dimensional imprint on bystander resuscitation—reshaping the bystander CPR rate trajectory asymmetrically by arrest location while shifting the CPR method uniformly toward compression-only CPR—with bystander AED use and short-term outcomes unchanged. First, the pre-COVID-19 rising bystander CPR trend slowed in public and reversed at home (cumulative counterfactual shortfalls ≈18 and 12 pp); the location asymmetry was directionally consistent across robustness specifications, significant in witnessed arrests (*p* = 0.046) and marginal in the full adjusted model (*p* = 0.065), with a three-segment model suggesting partial endemic recovery at home. Second, the method of CPR shifted abruptly and uniformly toward compression-only CPR, with conventional CPR collapsing from 7–9% to <3% of bystander-performed CPR; directionally consistent across pre-period sensitivity analyses, although the level-change magnitude was partly leveraged by the 2018 conventional CPR peak. Third, bystander AED use was unchanged. Fourth, formal period × location interaction tests showed no differential change in ROSC, survival or good neurological outcome (all non-significant).

These findings extend the literature. Lim et al. examined only public-location arrests in January–June 2020 and reported no change [12]; our 2016–2025 ITS confirms the absent immediate level change in public but identifies a home slope change and partial endemic recovery that a single-period analysis cannot detect. Acute-pandemic reports from France, Italy and the United States reported aggregate decreases without separating location or method [13,14,15], as did an Osaka City report [21]. A worldwide multi-registry analysis confirmed lockdown-associated reductions in bystander CPR but did not stratify by location or method [16], and a 2025 meta-analysis confirmed reduced bystander CPR across diverse settings [17]. Magnitude tracked local pandemic burden and health system capacity: severely affected settings saw concurrent declines in bystander CPR and survival (Osaka, Japan; Telangana, India) [22,23], a German registry found the rate stable but survival lower amid a shift toward home [24], and a nationwide Japanese cohort found no deterioration [25], consistent with our preserved outcomes. Our specific contribution is to test, in a national registry ITS framework, the dual hypothesis that COVID-19 would affect bystander CPR rate differently between home and public settings (location-asymmetric, partially supported) and shift the method uniformly toward compression-only CPR (supported), providing setting-specific evidence that international meta-analyses cannot resolve.

Mechanistically, the abrupt method shift is biologically plausible. Mouth-to-mouth ventilation was widely identified as a transmission risk during the acute pandemic, and major resuscitation councils issued interim guidance favouring compression-only CPR for untrained or potentially exposed bystanders [7,8]. Behavioural surveys provide direct evidence that lay bystanders de-prioritised rescue breathing during the pandemic. In a Canadian survey, willingness to give rescue breaths to a stranger fell from 66.5% to 51.0% during the pandemic (*p* < 0.001)—the largest decline among surveyed CPR actions, exceeding the fall in willingness to perform chest compressions [26]. In a Taiwanese survey, 40.1% of those reluctant to perform conventional bystander CPR would still act if it could be hands-only, and respondents preferred CPR without rescue breathing [27]. Consistent with this mechanism, some systems modified dispatcher-assisted CPR to add mouth-and-nose covering before compressions, leaving the resuscitation rate unchanged but delaying its initiation [28]. The decisive observation against a purely secular interpretation is the temporal profile: conventional CPR showed no significant pre-COVID-19 trend (OR 1.034/quarter; *p* = 0.31), then dropped abruptly and significantly at the interruption (OR 0.22; *p* = 0.030) with no recovery, concurrently in both settings. Such a pattern does not fit the gradual, guideline-driven move toward compression-only CPR of the previous decade, which would have produced a slow decline rather than an abrupt drop that began in the same month, was of almost identical size at home and in public, and never reversed; the behavioural survey evidence instead points to a COVID-19-specific reluctance to give rescue breaths as the immediate cause. Importantly, this method shift did not translate into fewer bystanders acting: decomposing the response into no CPR, compression-only CPR and conventional CPR showed that the collapse of conventional CPR was absorbed by compression-only rather than by an increase in no bystander CPR (Supplementary Figure S8), indicating method substitution rather than a loss of responders and consistent with the preserved short-term outcomes.

The rate divergence has a separate, more tentative interpretation. During the pandemic, OHCA shifted toward home, plausibly making family bystanders the dominant rescuer pool; the ancillary KoCARC relationship variable, discontinued after 2018, was, based on pre-pandemic evidence, consistent with family witnesses dominating at home (94%) and lay strangers in public (69%). This interpretation is speculative and cannot be tested directly, because the relationship variable was unavailable after 2018; on this account, the cumulative home shortfall might reflect family rescuers’ greater sensitivity to lockdown anxiety, isolation and reduced public CPR exposure, whereas public locations retained device infrastructure and trained-bystander resilience. The asymmetry was concentrated in witnessed arrests (*p* = 0.046 versus *p* = 0.40 in unwitnessed)—the subgroup with the clearest bystander CPR opportunity—supporting a behavioural rather than purely structural explanation. We caution, however, that the case mix shifted across periods (age 65.8→68.5 years; witnessed 57.9→61.7%) and that the relationship-proxy validation predates the pandemic; the adjusted full-cohort interaction was only marginally significant (*p* = 0.065), so a competing case-mix mechanism cannot be excluded. Moreover, a placebo interruption one year before COVID-19 detected a larger level break in the home rate series (January 2019; *p* < 0.001; Supplementary Table S3) than at the true interruption, indicating pre-COVID-19 structural instability specific to the home series; the home slope reversal should therefore be read as hypothesis-generating. This caveat applies to the rate finding only: the method shift is abrupt, concurrent and near-identical across settings and is unaffected by this placebo signal. The nursing facility stratum, where bystander CPR was high and showed no material change across periods (67.9%, 70.0%, 69.1%; overlapping Wilson confidence intervals; Supplementary Table S6), is consistent with the family rescuer interpretation in that trained staff were less exposed to lockdown behavioural effects.

For policy, our findings document a COVID-19-era acceleration of a known trajectory rather than identifying a new direction. Korean and Asian guidelines have promoted compression-only CPR since the 2010s [29], and the >97% compression-only share we observe in 2023–2025 essentially closes the conventional CPR gap; given non-inferiority for adult cardiac-origin OHCA [30,31], national training can consolidate around compression-only CPR for lay bystanders. Notably, the compression-only share did not recede after the pandemic eased but persisted through the endemic period, suggesting that the COVID-19-era shift became entrenched as a durable learning and habituation effect rather than a transient infection control response. Because this near-complete disappearance of bystander rescue breathing could disadvantage arrests in which ventilation is important—asphyxial and respiratory-origin arrest, drowning, paediatric arrest and prolonged resuscitation—conventional (compression-plus-ventilation) CPR training should be maintained and reinforced for trained responders and these specific scenarios, so that the capability to deliver rescue breaths is preserved where it is most needed. The home shortfall reinforces existing international recommendations [6,32,33] that residential rapid activation (dispatcher-assisted CPR, smartphone-activated volunteer responders, family-targeted CPR training) is a priority target; system-level initiatives produce durable improvements in bystander intervention and survival in other registries [34]. Bystander AED use was uniformly low and unchanged across periods in both settings (0.5–0.6% at home versus 3.8–4.9% in public), mirroring Korea’s persistently low national utilisation of roughly 1–2% of OHCA over the past decade despite dense device installation [4,35]; differential public-access defibrillation is therefore an unlikely explanation for the location-asymmetric rate change, although unchanged AED use does not exclude the broader structural advantage of public settings (trained and professional first responders, higher witness density), which remains an alternative to the family rescuer account. Nonetheless, bystander AED, when applied, is associated with markedly improved outcomes [36]; so, the unchanged low residential AED rate identifies an enduring unmet opportunity at home. Dispatcher-assisted CPR—a key mediator of residential rapid activation in Korea—could not be analysed here because of >95% missingness; because dispatcher assistance is particularly associated with bystander CPR in residential arrests, its omission may be especially relevant to the home-specific rate findings, and unmeasured pandemic-era changes in dispatcher recognition or protocols could themselves have contributed to the location-asymmetric trajectory, and future evaluation will require dispatcher-linked datasets.

### Limitations

First, KoCARC enrols OHCAs transported to 33 predominantly tertiary urban hospitals, limiting rural and field-terminated generalisability. Second, Korea’s lockdown stringency was substantially milder than in many Western jurisdictions, without the prolonged, mandatory stay-at-home orders imposed elsewhere; the magnitude of our findings therefore cannot be directly compared with countries that enacted severe lockdowns, where the behavioural and system-level disruption to bystander CPR is likely to have been considerably greater, and our estimates may understate the pandemic’s impact in those settings. Third, the binary public–home dichotomy conceals within-stratum heterogeneity [37]. Fourth, the bystander relationship variable was an optional KoCARC field whose collection collapsed after 2018, so our family-versus-stranger interpretation rests on a pre-pandemic proxy (Supplementary Figure S5) and the pandemic may have changed who was at home during an OHCA. Fifth, the ITS quasi-experimental design cannot establish causation; a placebo interruption test detected a significant level break at home in January 2019 (*p* < 0.001), indicating a pre-COVID-19 structural shift, and the conventional CPR level break lost statistical significance when 2018 was excluded; so, the headline findings must be interpreted as directional and consistent rather than as robust point effects, and counterfactual extrapolations through 2025 are model-dependent. Sixth, dispatcher-assisted CPR (>95% missingness) could not be analysed. Seventh, COVID-19-era respiratory-origin OHCA may be misclassified as “medical aetiology”, dispatcher recognition and field termination thresholds may have shifted during the pandemic, and the registry captures only transported cases—all unmeasured selection mechanisms. The ~14% of arrests with unclassified location that were excluded were, moreover, not evenly distributed across periods (13.9%, 13.3%, 15.0%; chi-square *p* = 0.010; Supplementary Table S4), so differential exclusion may have contributed to the location-specific trajectories; excluded undefined-location cases also differed systematically from included cases (more often witnessed and shockable, a lower bystander CPR rate, and higher survival; Supplementary Table S8) and showed their own pandemic-era level change in bystander CPR (Supplementary Table S9), so this exclusion is not innocuous. Eighth, primary analyses were defined a priori internally but not publicly pre-registered; multiplicity was not formally adjusted.

## 5. Conclusions

In a multicentre Korean OHCA cohort spanning 2016–2025, COVID-19 was associated with an abrupt, uniform shift in bystander CPR toward compression-only CPR (>97%; directionally consistent across pre-period sensitivity) and a more limited and suggestive location-asymmetric change in the bystander CPR rate (a slowed rise in public and a decline at home; significant only in witnessed arrests and marginal in the full adjusted analysis, and therefore hypothesis-generating rather than definitive), with no period × location effect on short-term outcomes. The findings reinforce existing recommendations to consolidate lay-bystander training around compression-only CPR and to prioritise residential rapid activation infrastructure.

## Figures and Tables

**Table 1:** Patient characteristics, bystander CPR and clinical outcomes by COVID-19 period.

Characteristic	Pre-COVID-19 (2016–January 2020)	Pandemic (February 2020–May 2023)	Endemic (June 2023–2025)	*p* Value
No. of patients	8817	8477	3888	
Age, mean (SD)	65.8 (18.6)	67.3 (17.5)	68.5 (17.4)	<0.001
Male sex, *n* (%)	5788 (65.6)	5794 (68.3)	2545 (65.5)	<0.001
Home (private), *n* (%)	6677 (75.7)	6569 (77.5)	2982 (76.7)	0.023
Public, *n* (%)	2140 (24.3)	1908 (22.5)	906 (23.3)	0.023
Witnessed arrest, *n* (%)	5031 (57.9)	4976 (58.8)	2393 (61.7)	<0.001
Shockable rhythm, *n* (%)	1642 (19.8)	1445 (17.5)	654 (17.4)	<0.001
Any bystander CPR, *n* (%)	4558 (54.5)	4800 (58.1)	2132 (56.2)	<0.001
Bystander CPR method				
Compression-only, *n* (%)	4224 (92.7)	4677 (97.4)	2086 (97.8)	<0.001
Conventional (+rescue breathing), *n* (%)	334 (7.3)	123 (2.6)	46 (2.2)	<0.001
Bystander AED used, *n* (%)	109 (1.3)	110 (1.3)	61 (1.6)	0.343
Prehospital ROSC, *n* (%)	1238 (14.0)	1131 (13.3)	608 (15.6)	0.003
Survival to discharge, *n* (%)	1128 (12.8)	1012 (11.9)	492 (12.7)	0.209
Good neurological outcome (CPC 1-2), *n* (%)	782 (8.9)	672 (7.9)	323 (8.3)	0.081

*SD, standard deviation. p values from Kruskal–Wallis (continuous) or chi-square (categorical) across the three periods. Compression-only and conventional CPR are percentages of bystander-performed CPR. The bystander CPR rate and witnessed-arrest proportion are calculated among cases with known status, whose denominators are smaller than the tabulated number of patients; the listed percentages therefore may not reproduce exactly from the cohort counts. Exact numerators and denominators for every variable and period are given in Supplementary Table S7.*

**Table 2:** Interrupted time-series segmented regression of any bystander CPR rate (monthly).

Parameter	Overall	Home	Public
Pre-COVID-19 trend (pp/month)	0.197 (0.118 to 0.276); *p* < 0.001	0.185 (0.093 to 0.277); *p* < 0.001	0.236 (0.121 to 0.352); *p* < 0.001
Level change at COVID (pp)	0.005 (−2.962 to 2.973); *p* = 0.997	0.496 (−3.036 to 4.027); *p* = 0.783	−2.668 (−6.991 to 1.656); *p* = 0.227
Trend change (pp/month)	−0.253 (−0.351 to −0.154); *p* < 0.001	−0.278 (−0.394 to −0.163); *p* < 0.001	−0.151 (−0.284 to −0.019); *p* = 0.025
Post-COVID-19 net trend (b1 + b3)	−0.056 pp/month	−0.093 pp/month	+0.085 pp/month

*Data are estimates (95% CI); p value. Segmented regression with Newey–West HAC standard errors and annual harmonic terms; interruption point February 2020. Baseline bystander CPR levels in 2016 were 49.7% (overall), 50.9% (home) and 46.0% (public); the modelled effect at June 2025 versus counterfactual was −16.4, −17.6 and −12.5 pp. Pre-COVID-19 trend = β1; immediate level change at the interruption = β2; trend (slope) change = β3; post-COVID-19 net trend = β1 + β3. Effect at 2025 = observed minus counterfactual (baseline level + β1·time extrapolated through June 2025). CI, confidence interval; CPR, cardiopulmonary resuscitation; HAC, heteroscedasticity- and autocorrelation-consistent; pp, percentage point.*

**Table 3:** Interrupted time-series of conventional (rescue breathing) bystander CPR share.

Parameter	Overall	Home	Public
Pre-COVID-19 trend (OR/quarter)	1.034 (0.969–1.102); *p* = 0.312	1.039 (0.973–1.109); *p* = 0.250	1.020 (0.945–1.102); *p* = 0.606
Level change at COVID-19 (OR)	0.221 (0.057–0.863); *p* = 0.030	0.212 (0.052–0.858); *p* = 0.030	0.264 (0.073–0.957); *p* = 0.043
Trend change (OR/quarter)	0.979 (0.888–1.079); *p* = 0.672	0.982 (0.893–1.081); *p* = 0.716	0.961 (0.854–1.081); *p* = 0.510
Level change, absolute (pp; quarterly OLS)	−6.6 pp (−10.4 to −2.8); *p* < 0.001	−6.5 pp (−10.4 to −2.6); *p* = 0.001	−6.9 pp (−10.9 to −2.9); *p* < 0.001
Effect at 2025 (vs. counterfactual)	−10.1 pp	−9.7 pp	−11.2 pp

*Data are odds ratio (95% CI); p value, except the absolute level-change row (percentage points from an aggregated quarterly OLS model). Individual-level segmented logistic regression with hospital-cluster robust standard errors; interruption point 2020Q1. Pre-COVID-19 trend, secular trend before the interruption; level change, immediate change at the interruption; trend change, change in slope thereafter. Compression-only CPR is the complement of conventional CPR among performed cases (mirror analysis in Figure 4 and Supplementary Figure S7). CI, confidence interval; CPR, cardiopulmonary resuscitation; OLS, ordinary least squares; OR, odds ratio; pp, percentage point.*

## Data Availability

The KoCARC data are available on reasonable application to the consortium; the analytic Python code is available from the corresponding author on reasonable request.

## Supplementary Materials

The following supporting information can be downloaded at: https://www.mdpi.com/article/10.3390/jcm15145469/s1, Figure S1: Arrest-location mix shifted toward home during the pandemic (75.7% → 77.5% → 76.7%; *p* = 0.023); Figure S2: Descriptive bystander CPR rate by location and period (3-group bars with Wilson 95% CI); complements the Figure 3 ITS; Figure S3: Bystander AED use by location and period; no significant period change in either setting; Figure S4: CPR-type composition (compression-only vs conventional vs not performed) within each location × period stratum; Figure S5: Where recorded, the bystander–patient relationship validates arrest location as a relationship proxy (family witnesses 94% at home, layperson witnesses 69% in public); relationship-stratified bystander CPR rates by relationship category; Figure S6: ITS of conventional CPR share (Overall, Home, Public)—same abrupt level drop at COVID-19 interruption in all three strata; Figure S7: Mirror view: ITS of compression-only CPR share—abrupt level jump at COVID-19 interruption, sustained at >97% in all three strata; Figure S8: Three-level composition of the bystander response (no bystander CPR, compression-only, conventional) as a percentage of all OHCA with known bystander-CPR status: (a) annual trajectory 2016–2025; (b) by COVID-19 period; Table S1: Definition of study periods; Table S2: Adjusted individual-level segmented logistic regression for the any-bystander-CPR rate, with location × interruption interaction; Table S3: Interrupted time-series robustness battery for the any-bystander-CPR rate (cut-point, 3-segment, placebo, bootstrap, and HAC/harmonic sensitivity); Table S4: Method, witnessed-stratified, and Other/Unknown sensitivity analyses; Table S5: Adjusted outcome models with period × location interaction; Table S6: Nursing-facility stratum: bystander CPR rate by COVID-19 period; Table S7: Exact numerators and denominators by period (main cohort, Home + Public); Table S8: Comparison of included (classified location) and excluded (undefined location) cases; Table S9: Sensitivity analysis treating unknown location as a separate category; Table S10: Weighting (OLS vs n-weighted WLS) and time-series diagnostics for the any-CPR rate ITS; Table S11: Conventional-CPR interruption-coding sensitivity; Table S12: Outcome period × location interaction, with vs without mediator adjustment; Checklist S1: STROBE Statement—checklist of items that should be included in reports of observational studies.

## Abbreviations

OHCA, out-of-hospital cardiac arrest; CPR, cardiopulmonary resuscitation; AED, automated external defibrillator; ITS, interrupted time-series; ROSC, return of spontaneous circulation; CPC, Cerebral Performance Category; KoCARC, Korean Cardiac Arrest Research Consortium.
