# Supplementary material for: Changes in Bystander CPR Patterns of Private and Public Locations Before and After COVID-19: An Interrupted Time-Series of a Multicentre Out-of-Hospital Cardiac Arrest Cohort
Source: J Clin Med. 2026 Jul 13;15(14):5469. doi: 10.3390/jcm15145469 (PMC13411539; doi:10.3390/jcm15145469)
Supplement: Supplementary file 1 [file jcm-15-05469-s001.zip › jcm-4387019-Checklist S1.pdf]

Checklist S1: STROBE Statement - Checklist of items for cohort studies

Manuscript: "Changes in Bystander CPR Patterns of Private and Public Locations before and after COVID-19: An Interrupted Time-Series of a Multicentre Out-of-Hospital Cardiac Arrest Cohort"

Study design: Observational cohort study (retrospective analysis of a prospectively collected multicentre registry), analysed with an interrupted time-series (ITS) design. Reported per the STROBE statement, supplemented by Bernal, Cummins & Gasparrini (Int J Epidemiol 2017) ITS reporting guidance.

Section / Topic	Item #	STROBE recommendation	Location in manuscript
**Title and abstract**	1 (a)	Study design indicated in the title	Title: "...An Interrupted Time-Series of a Multicentre Out-of-Hospital Cardiac Arrest Cohort."
	1 (b)	Balanced summary in the abstract	Abstract (Background / Methods / Results / Conclusions), structured.
**Introduction**			
**Background/rationale**	2	Scientific background and rationale	Introduction (competing pandemic pressures on bystander CPR; gaps by location and CPR method).
**Objectives**	3	Specific objectives / hypotheses	Introduction, final paragraph (co-primary objectives: any bystander CPR; conventional vs compression-only CPR by location).
**Methods**			
**Study design**	4	Key elements of design early in paper	Methods, "Study design and data source"; interrupted time-series with segmented regression.
**Setting**	5	Setting, locations, relevant dates	Methods, "Study design and data source" and "Period stratification": KoCARC, 33 hospitals, Korea; October 2015-June 2025; Pre-COVID / Pandemic / Endemic periods.
**Participants**	6 (a)	Eligibility criteria, sources, methods of selection; follow-up	Methods, "Study design and data source": adults (>=18 y) OHCA of presumed medical aetiology; eligibility window; exclusions. Figure 1 (flow).

	6 (b)	Matched studies: matching criteria	Not applicable (no matched design).
**Variables**	7	Outcomes, exposures, predictors, confounders, effect modifiers	Methods, "Outcomes", "Location classification", "Bystander CPR method classification"; confounders in multivariable models (age, sex, witnessed, shockable rhythm, bystander CPR, bystander AED).
**Data sources/measurement**	8	Sources and methods of assessment for each variable	Methods, "Study design and data source": standardised web-based case report form following the Utstein template.
**Bias**	9	Efforts to address potential sources of bias	Methods (a priori analysis plan; HAC / cluster-robust SE; sensitivity strata) and Discussion (limitations: selection toward tertiary urban hospitals).
**Study size**	10	How study size was arrived at	Results, "Cohort" and Figure 1: of 26,589 registry cases, 21,182 formed the main cohort after specified exclusions.
**Quantitative variables**	11	Handling of quantitative variables; groupings	Methods, "Period stratification" (3 periods), "Location classification" (private vs public), "Bystander CPR method classification" (3 mutually exclusive categories).
**Statistical methods**	12 (a)	Methods including control of confounding	Methods, "Statistical analysis": segmented regression with harmonics; Newey-West HAC SE; individual-level segmented logistic regression with hospital-cluster robust SE.
	12 (b)	Examination of subgroups and interactions	Methods / Results: period x location interaction tests; analyses stratified by location.
	12 (c)	How missing data were addressed	Methods, "Statistical analysis" / Supplement (analysis restricted to classified bystander-CPR status; handling described).
	12 (d)	Loss to follow-up / sampling addressed	Not applicable (event-level registry outcomes;

			no longitudinal loss to follow-up).
	12 (e)	Sensitivity analyses	Methods / Results and Supplement: nursing-facility stratum (Table S6); block-bootstrap CIs; additional sensitivity analyses.
**Results**			
**Participants**	13 (a-c)	Numbers at each stage; non-participation; flow diagram	Results, "Cohort"; Figure 1 (study flow diagram).
**Descriptive data**	14 (a-c)	Characteristics; missing data; follow-up time	Results, "Cohort"; Table 1 (baseline characteristics by period).
**Outcome data**	15	Number of outcome events / summary measures	Results ("Bystander CPR rate", "Bystander CPR method", "Bystander AED use", "Outcomes"); Tables 1-3.
**Main results**	16 (a-c)	Estimates, CIs, confounder adjustment; category boundaries; absolute risk	Results; Tables 2-3 and Figures 2-5: ITS level/slope changes with 95% CIs; ORs with 95% CIs.
**Other analyses**	17	Subgroups, interactions, sensitivity analyses	Results, "Outcomes" (period x location interaction); Supplement (Tables/Figures S1-S6).
**Discussion**			
**Key results**	18	Summary with reference to objectives	Discussion, opening paragraph.
**Limitations**	19	Limitations, sources of bias, direction/magnitude	Discussion, limitations paragraph(s).
**Interpretation**	20	Cautious overall interpretation	Discussion.
**Generalisability**	21	External validity	Discussion, limitations (tertiary urban hospitals; milder lockdown stringency vs other countries).
**Other information**			
**Funding**	22	Funding source and role	Funding statement: "This research received no external funding."

Note: CONSORT 2025 (randomised controlled trials) and TREND (non-randomised interventional trials) are not applicable to this study, which is observational (no investigator-allocated intervention; ClinicalTrials.gov Study Type "Observational", NCT03222999). For the interrupted time-series component, reporting also follows Bernal JL, Cummins S, Gasparrini A. Interrupted time series regression for the evaluation of public health interventions: a tutorial. Int J Epidemiol 2017;46(1):348-355.
