# Supplementary material for: Changes in Bystander CPR Patterns of Private and Public Locations Before and After COVID-19: An Interrupted Time-Series of a Multicentre Out-of-Hospital Cardiac Arrest Cohort
Source: J Clin Med. 2026 Jul 13;15(14):5469. doi: 10.3390/jcm15145469 (PMC13411539; doi:10.3390/jcm15145469)

Supplementary Materials

Changes in Bystander CPR Patterns of Private and Public Locations before and after COVID-19: An Interrupted Time-Series of a Multicentre Out-of-Hospital Cardiac Arrest Cohort

Table S1. Definition of study periods.

Period	Calendar span	Policy basis	Months	n (Home + Public)
Pre-COVID	January 2016 – January 2020	Before the KCDC national alert (26 January 2020)	49	8,817
Pandemic	February 2020 – May 2023	Highest national alert (23 February 2020) to the endemic transition (announced 11 May 2023)	40	8,477
Endemic	June 2023 – June 2025	After the crisis level was downgraded to alert (effective 1 June 2023)	25	3,888

Periods were assigned by month of arrest and aligned to Korea's COVID-19 policy timeline, so the descriptive boundaries coincide with the interrupted time-series break points (interruption February 2020; second knot May 2023).

Table S2. Adjusted individual-level segmented logistic regression for the any-bystander-CPR rate, with location × interruption interaction.

Cohort: n=20,332; hospitals=33 (cluster-robust SE).
Joint Wald for location × post + location × time-since-post interaction: W=5.46, df=2, P=0.06537.

Term	OR (95% CI)	P
Intercept	1.628 (1.309–2.026)	<0.001
Time (per month, Pre-COVID slope)	1.008 (1.004–1.013)	<0.001
Level change at COVID (Feb 2020), Home	1.014 (0.847–1.212)	0.883
Slope change post-COVID, Home	0.988 (0.982–0.993)	<0.001
Public location (vs Home)	0.798 (0.674–0.946)	0.009
Public × post (location-asymmetric level change)	0.933 (0.687–1.268)	0.659
Public × time-since-post (location-asymmetric slope change)	1.007 (1.000–1.015)	0.059
sin(2πt/12)	1.035 (0.979–1.094)	0.223
cos(2πt/12)	0.984 (0.942–1.028)	0.477
Age (per year)	0.992 (0.990–0.994)	<0.001
Male sex	0.973 (0.914–1.037)	0.401
Witnessed arrest	1.186 (1.081–1.300)	<0.001

Table S3. Interrupted time-series robustness battery for the any-bystander-CPR rate (cut-point, 3-segment, placebo, bootstrap, and HAC/harmonic sensitivity).

1. Cut-point sensitivity (slope change β_3, pp/month)

Stratum	Jan 2020	Feb 2020 (primary)	Mar 2020	Apr 2020
Home	-0.293 (P<0.001)	-0.278 (P<0.001)	-0.278 (P<0.001)	-0.285 (P<0.001)
Public	-0.158 (P=0.026)	-0.151 (P=0.025)	-0.136 (P=0.037)	-0.132 (P=0.037)
Overall	-0.266 (P<0.001)	-0.253 (P<0.001)	-0.250 (P<0.001)	-0.255 (P<0.001)

2. 3-segment ITS (second break May 2023 = Korean endemic declaration)

Stratum	Pre-COVID trend	Pandemic slope change	Endemic slope change	Net pre / pandemic / endemic trend
Home	+0.184 (P<0.001)	-0.382 (P<0.001)	+0.241 (P=0.137)	+0.184 / -0.199 / +0.043
Public	+0.236 (P<0.001)	-0.070 (P=0.437)	-0.005 (P=0.976)	+0.236 / +0.167 / +0.161
Overall	+0.196 (P<0.001)	-0.316 (P<0.001)	+0.175 (P=0.205)	+0.196 / -0.119 / +0.056

3. Placebo interruption tests

Significant level/slope changes should NOT appear at false interruption points if the true effect is COVID-specific.

Stratum	Jan 2018 placebo	Jan 2019 placebo	Feb 2020 true
Home	lvl -0.36 (P=0.833); slope -0.276 (P=0.035)	lvl -5.70 (P<0.001); slope +0.271 (P=0.072)	lvl +0.50 (P=0.783); slope -0.278 (P<0.001)
Public	lvl +0.36 (P=0.908); slope +0.157 (P=0.440)	lvl +3.35 (P=0.447); slope -0.250 (P=0.629)	lvl -2.67 (P=0.227); slope -0.151 (P=0.025)
Overall	lvl +0.18 (P=0.910); slope -0.187 (P=0.115)	lvl -3.97 (P=0.008); slope +0.167 (P=0.424)	lvl +0.01 (P=0.997); slope -0.253 (P<0.001)

4. Counterfactual gap at series end (Jun 2025) — bootstrap 95% CI

Block-bootstrap (block size 6 months) over Newey-West regression residuals, B=2,000 replications.

Stratum	Counterfactual gap (pp)	95% CI
Home	-17.60	-26.53 to -9.12
Public	-12.50	-23.14 to -2.66
Overall	-16.42	-23.76 to -9.01

5. HAC lag + semi-annual harmonics sensitivity (slope change β_3)

Stratum	Specification	β_3 (pp/month)	P
Home	annual, maxlag=4	-0.2784	<0.001
Home	annual, maxlag=6	-0.2784	<0.001
Home	annual, maxlag=12	-0.2784	<0.001
Home	annual, maxlag=18	-0.2784	<0.001
Home	annual+semi, maxlag=4	-0.2776	<0.001
Public	annual, maxlag=4	-0.1513	0.025
Public	annual, maxlag=6	-0.1513	0.010

Public	annual, maxlag=12	-0.1513	<0.001
Public	annual, maxlag=18	-0.1513	<0.001
Public	annual+semi, maxlag=4	-0.1488	0.031
Overall	annual, maxlag=4	-0.2528	<0.001
Overall	annual, maxlag=6	-0.2528	<0.001
Overall	annual, maxlag=12	-0.2528	<0.001
Overall	annual, maxlag=18	-0.2528	<0.001
Overall	annual+semi, maxlag=4	-0.2516	<0.001

Table S4. Method, witnessed-stratified, and Other/Unknown sensitivity analyses.

A. Conventional CPR — pre-period exclusion sensitivity

Individual-level segmented logistic regression, hospital-cluster robust SE. Quarterly time variable.

Scenario	n	Pre-COVID trend OR/qtr	Level change OR	Slope change OR/qtr
All years 2016–2025 (primary)	11,490	1.034 (0.969–1.102); P=0.312	0.223 (0.057–0.872); P=0.031	0.979 (0.888–1.079); P=0.664
Exclude 2018	10,262	1.021 (0.953–1.093); P=0.556	0.299 (0.083–1.074); P=0.064	0.991 (0.895–1.097); P=0.861
Exclude 2018–2019	9,050	1.071 (0.862–1.330); P=0.535	0.171 (0.008–3.534); P=0.253	0.945 (0.763–1.170); P=0.601
Post-2018 only	8,231	1.041 (0.622–1.743); P=0.879	0.289 (0.067–1.245); P=0.096	0.973 (0.572–1.654); P=0.918

B. Witnessed-stratified any-CPR ITS

Subgroup	n	Joint Wald W (df=2)	Joint P (location × post + location × time-since-post)
Witnessed	11,969	6.18	0.046
Unwitnessed	8,363	1.86	0.395

C. Other/Unknown location share by period

Tests whether the 14% Other/Unknown exclusion differed across COVID periods.

Period	N	n Other/Unknown	%
Endemic	6,085	912	14.99%
Pandemic	9,521	1,263	13.27%
Pre-COVID	10,711	1,488	13.89%

chi-square (period × Other vs not): 9.20, P=0.010.

Table S5. Adjusted outcome models with period × location interaction.

Multivariable logistic regression with period (Pre-COVID/Pandemic/Endemic) × location (Home/Public) interaction, adjusted for age, sex, witnessed arrest, shockable initial rhythm, bystander CPR (any), and bystander AED. Hospital-cluster robust standard errors.

Outcome	n	Pandemic × Public OR (95% CI)	Endemic × Public OR (95% CI)	Joint LR(2) P
Prehospital ROSC	19,366	0.85 (0.68–1.07); P=0.171	1.08 (0.80–1.46); P=0.607	3.45; P=0.178
Survival to discharge	19,366	1.10 (0.88–1.36); P=0.402	1.17 (0.90–1.51); P=0.245	1.25; P=0.536
Good neurological outcome (CPC 1–2)	19,366	0.98 (0.75–1.27); P=0.861	1.23 (0.88–1.72); P=0.217	1.88; P=0.391

Table S6. Nursing-facility stratum: bystander CPR rate by COVID-19 period.

Period	n/N	Bystander CPR rate (%)	95% CI
Pre-COVID (2016–Jan 2020)	390/574	67.9	64.0–71.6
Pandemic (Feb 2020–May 2023)	403/576	70.0	66.1–73.6
Endemic (Jun 2023–2025)	179/259	69.1	63.2–74.4

Table S7. Exact numerators and denominators by period (main cohort, Home + Public)

Each cell is events / cases with known status (percentage). Denominators differ across rows because not every case has a recorded value for every variable. CPR method percentages use bystander-performed CPR (compression-only + conventional) as the denominator; the bystander-CPR rate uses cases with known bystander-CPR status; outcomes use the full cohort (no missing).

Variable	Pre-COVID	Pandemic	Endemic
Any bystander CPR (rate)	4,558/8,370 (54.5%)	4,800/8,266 (58.1%)	2,132/3,796 (56.2%)
Conventional CPR (of performed)	334/4,558 (7.3%)	123/4,800 (2.6%)	46/2,132 (2.2%)
Compression-only (of performed)	4,224/4,558 (92.7%)	4,677/4,800 (97.4%)	2,086/2,132 (97.8%)
Witnessed arrest	5,031/8,696 (57.9%)	4,976/8,465 (58.8%)	2,393/3,880 (61.7%)
Bystander AED use	109/8,423 (1.3%)	110/8,317 (1.3%)	61/3,784 (1.6%)
Prehospital ROSC	1,238/8,817 (14.0%)	1,131/8,477 (13.3%)	608/3,888 (15.6%)
Survival to discharge	1,128/8,817 (12.8%)	1,012/8,477 (11.9%)	492/3,888 (12.7%)
Good neuro outcome (CPC1-2)	782/8,817 (8.9%)	672/8,477 (7.9%)	323/3,888 (8.3%)

Denominator availability (cases with known status / cohort N):

Status field	Pre-COVID	Pandemic	Endemic
Known bystander-CPR status	8,370/8,817 (94.9%)	8,266/8,477 (97.5%)	3,796/3,888 (97.6%)
Performed CPR (method denom)	4,558/8,817 (51.7%)	4,800/8,477 (56.6%)	2,132/3,888 (54.8%)
Known witnessed status	8,696/8,817 (98.6%)	8,465/8,477 (99.9%)	3,880/3,888 (99.8%)
Known bystander-AED status	8,423/8,817 (95.5%)	8,317/8,477 (98.1%)	3,784/3,888 (97.3%)

Table S8. Comparison of included (classified location) and excluded (undefined location) cases

Undefined location = KoCARC arrest-place codes 88/99/blank (n excludes nursing facilities, which were analysed as a separate stratum). Binary variables: events / known (percentage); P from chi-square (binary) or Welch t-test (age).

Variable	Included (Home+Public)	Excluded (undefined)	P
N	21,182	3,663	
Age, mean (SD)	66.9 (18.0)	65.6 (17.1)	<0.001
Male sex	14,127/21,182 (66.7%)	2,539/3,663 (69.3%)	0.002
Witnessed	12,400/21,041 (58.9%)	2,763/3,488 (79.2%)	<0.001
Shockable rhythm	3,741/20,298 (18.4%)	761/3,147 (24.2%)	<0.001
Any bystander CPR	11,490/20,432 (56.2%)	1,165/3,274 (35.6%)	<0.001
Bystander AED	280/20,524 (1.4%)	70/3,259 (2.1%)	<0.001
Prehospital ROSC	2,977/21,182 (14.1%)	634/3,663 (17.3%)	<0.001
Survival	2,632/21,182 (12.4%)	718/3,663 (19.6%)	<0.001
Good neuro	1,777/21,182 (8.4%)	487/3,663 (13.3%)	<0.001

Table S9. Sensitivity analysis treating unknown location as a separate category

(a) Stand-alone ITS of any-CPR rate in the undefined-location stratum (n=3,274): Pre-COVID trend +0.049 pp/month (P=0.655); level change -10.92 pp (P=0.010); trend change -0.018 pp/month (P=0.881).

Parameter	Estimate	P
Pre-COVID trend (pp/month)	+0.049	0.655
Level change at COVID interruption (pp)	-10.92	0.010
Trend change, post-COVID (pp/month)	-0.018	0.881

(b) Adjusted individual-level segmented logistic with 3-category location (Home reference / Public / Undefined), n=23,582: the Public-versus-Home interaction is unchanged from the main two-stratum model (joint Wald W=5.47, df=2, **P=0.065**). The undefined stratum instead showed a distinct, large immediate level drop (Undefined × post OR 0.50, P<0.001), so the full four-term joint Wald is significant (W=23.41, df=4, P<0.001). Excluding the undefined cases is therefore not innocuous — they behave differently from both classified strata, which reinforces the differential-exclusion limitation rather than the home-vs-public claim.

Term	Statistic	P
Public location × (post + t_post), joint Wald (cf. main two-stratum model)	W=5.47, df=2	0.065
Undefined location × post (immediate level change)	OR=0.50	<0.001
Full joint Wald, all location × interruption terms (Public + Undefined × post/t_post)	W=23.41, df=4	<0.001

Table S10. Weighting (OLS vs n-weighted WLS) and time-series diagnostics for the any-CPR rate ITS

The primary monthly model is unweighted OLS on the monthly proportion with Newey-West HAC standard errors and annual harmonics. As a sensitivity, the model was refitted as WLS with weights equal to the monthly number of arrests. The trend-change estimate (the key parameter) is essentially unchanged.

Stratum	OLS trend change (pp/month); P	WLS (n-weighted) trend change; P
Overall	-0.253; P=<0.001	-0.257; P=<0.001
Home	-0.278; P=<0.001	-0.281; P=<0.001
Public	-0.151; P=0.025	-0.166; P=0.016

Diagnostics (OLS residuals):

Overall: Durbin-Watson=1.84; Ljung-Box Q(12) P=0.066, Q(24) P=0.128; influential months (Cook's D>4/n): 6/114 (largest: 2020-01, 2016-01, 2016-05).

Home: Durbin-Watson=1.74; Ljung-Box Q(12) P=0.450, Q(24) P=0.241; influential months (Cook's D>4/n): 7/114 (largest: 2020-01, 2025-01, 2020-03).

Public: Durbin-Watson=2.12; Ljung-Box Q(12) P=0.393, Q(24) P=0.108; influential months (Cook's D>4/n): 5/114 (largest: 2024-04, 2016-01, 2016-05).

Newey-West HAC SEs already accommodate residual autocorrelation; the individual-level adjusted segmented logistic (Supplementary Table S2) provides the patient-weighted confirmation.

Table S11. Conventional-CPR interruption-coding sensitivity

In the quarterly model the interruption was coded at 2020Q1; because January 2020 belongs to the Pre-COVID period (the pandemic period starts February 2020), we refitted the individual-level segmented logistic with (a) a monthly time index and a February-2020 break (so January 2020 is correctly Pre-COVID), and (b) a quarterly model that drops the straddling 2020Q1 quarter.

Specification	n	Level-change OR (95% CI); P
Monthly, break at Feb 2020 (Jan 2020 = Pre-COVID)	11,490	0.198 (0.047-0.838); P=0.028
Quarterly, 2020Q1 excluded (break 2020Q2)	11,179	0.221 (0.047-1.029); P=0.054

Both specifications reproduce the abrupt level drop, confirming the finding is not an artefact of assigning January 2020 to the post-interruption segment.

Table S12. Outcome period x location interaction, with vs without mediator adjustment

Bystander CPR and bystander AED lie on the causal path between pandemic period/location and outcome, so they were re-classified as potential mediators. Models adjust for age, sex, witnessed arrest and shockable rhythm; the mediator set (bystander CPR, bystander AED) is added in the second column. Joint likelihood-ratio test (2 df) for the period x location interaction; hospital-cluster robust SE.

Outcome	LR(2) P, WITHOUT mediators	LR(2) P, WITH mediators
Prehospital ROSC	3.82; P=0.148	3.45; P=0.178
Survival to discharge	2.27; P=0.321	1.25; P=0.536
Good neurological outcome (CPC 1-	2.14; P=0.343	1.88; P=0.391

2)

The period x location interaction is non-significant in both specifications for all three outcomes, so the absence of a differential pandemic effect on outcomes is not an artefact of mediator adjustment.

Nursing-facility arrests (n=1,472) were analysed as a pre-specified sensitivity stratum, separate from the main home-versus-public contrast (1,409 of the 1,472 had known bystander-CPR status). Bystander CPR rate = chest compressions (with or without rescue breathing) among cases with known bystander-CPR status. CI, Wilson confidence interval.

Figure S1. Arrest-location mix shifted toward home during the pandemic (75.7% → 77.5% → 76.7%; P=0.023).

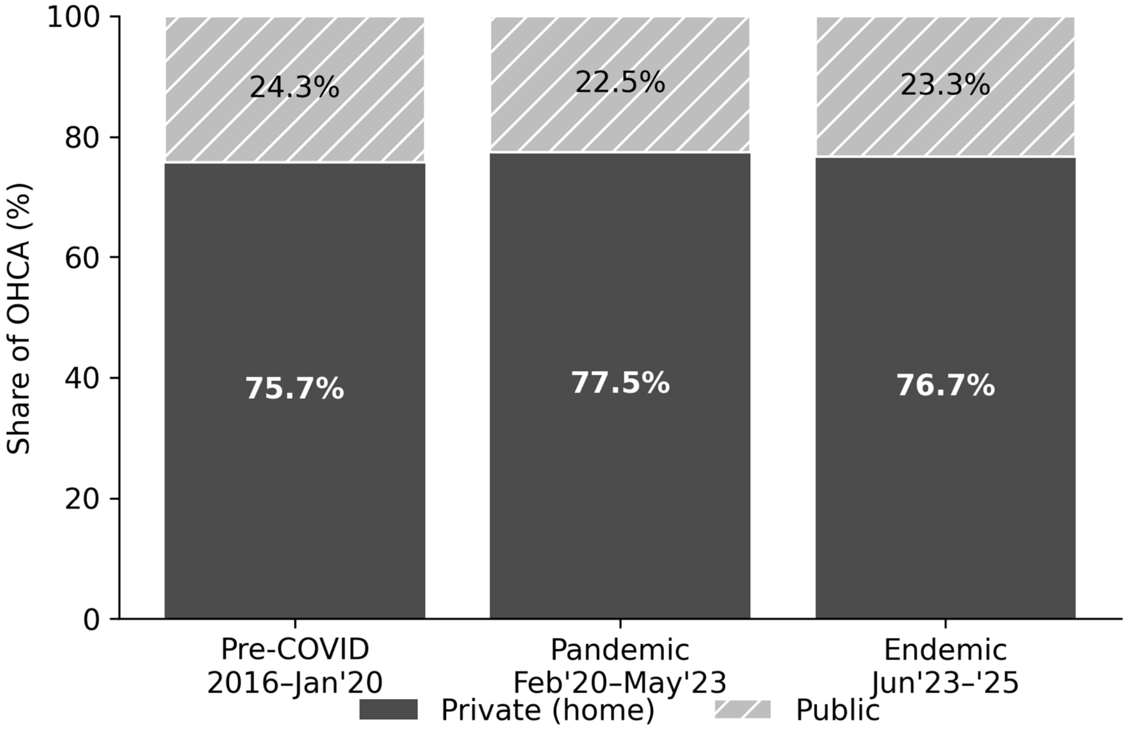

Figure S2. Descriptive bystander CPR rate by location and period (3-group bars with Wilson 95% CI); complements the Figure 3 ITS.

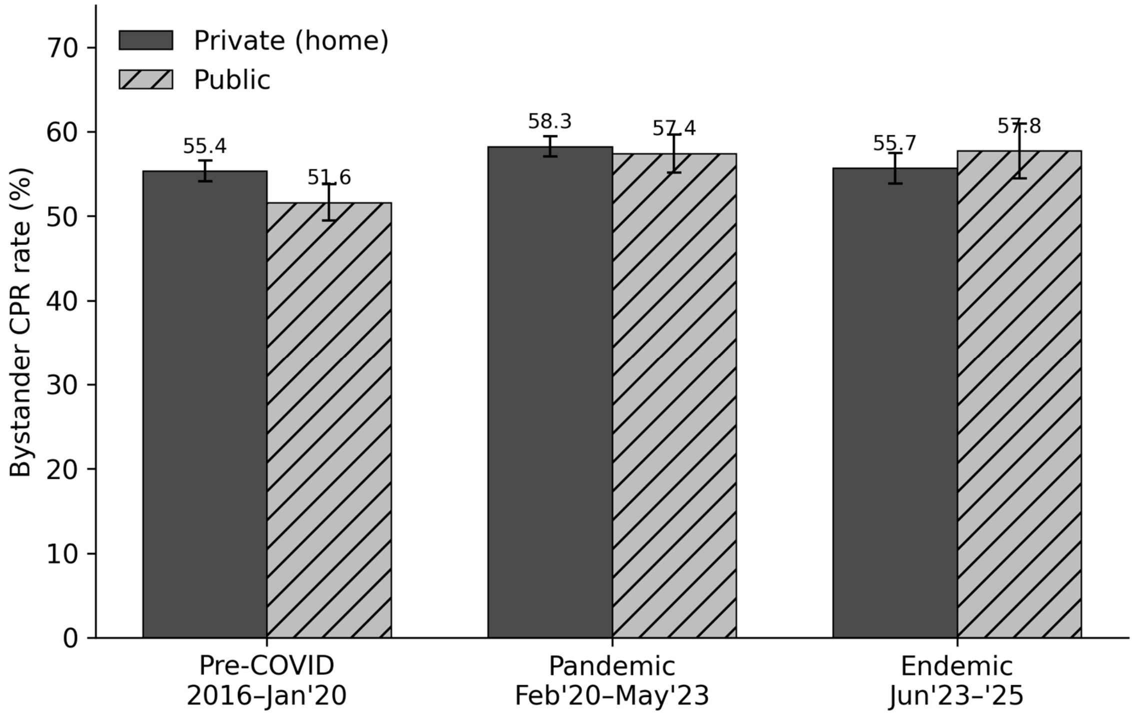

Figure S3. Bystander AED use by location and period; no significant period change in either setting.

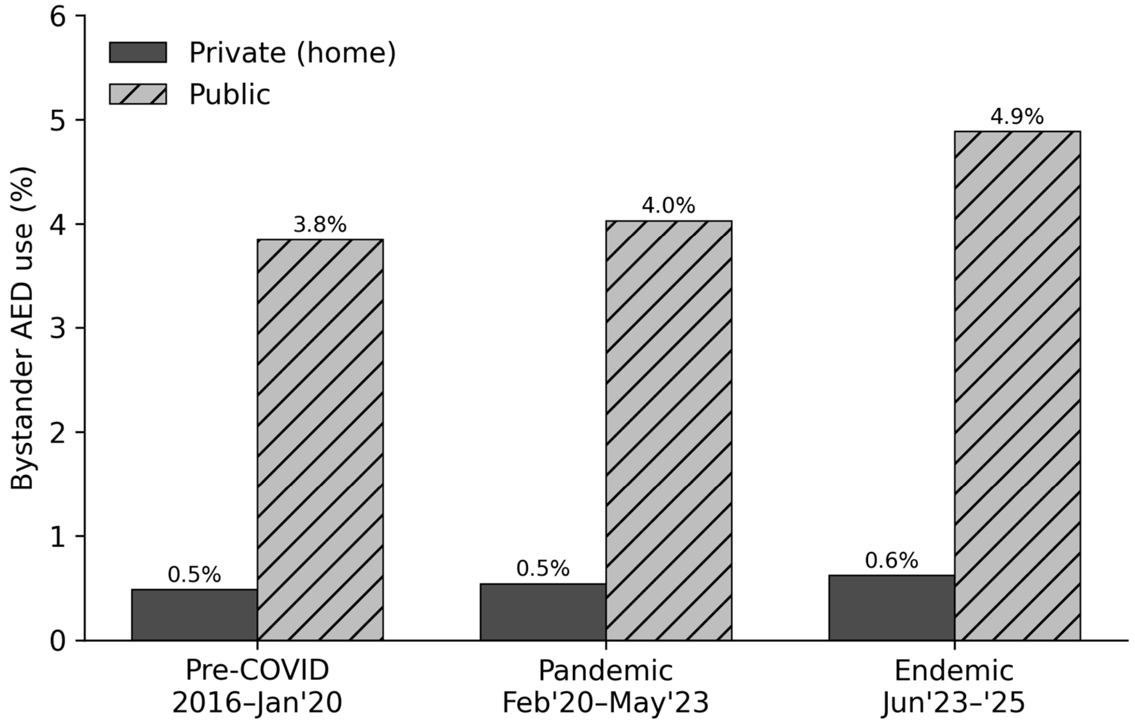

Figure S4. CPR-type composition (compression-only vs conventional vs not performed) within each location × period stratum.

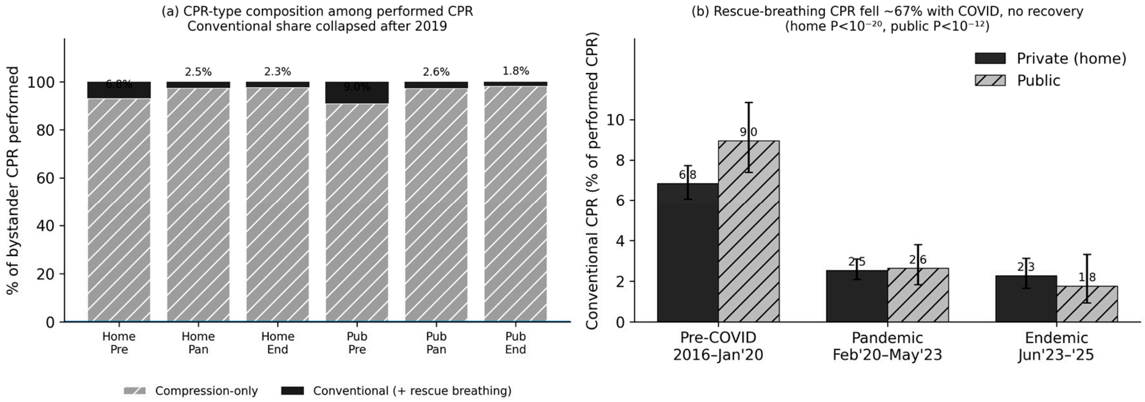

Figure S5. Where recorded, the bystander–patient relationship validates arrest location as a relationship proxy (family witnesses 94% at home, layperson witnesses 69% in public); relationship-stratified bystander CPR rates by relationship category.

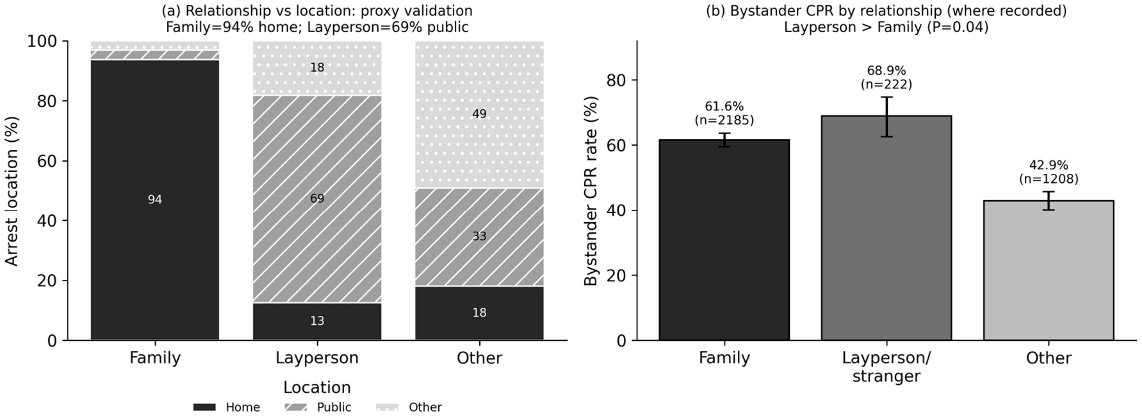

Figure S6. ITS of conventional CPR share (Overall, Home, Public) — same abrupt level drop at COVID in all three strata.

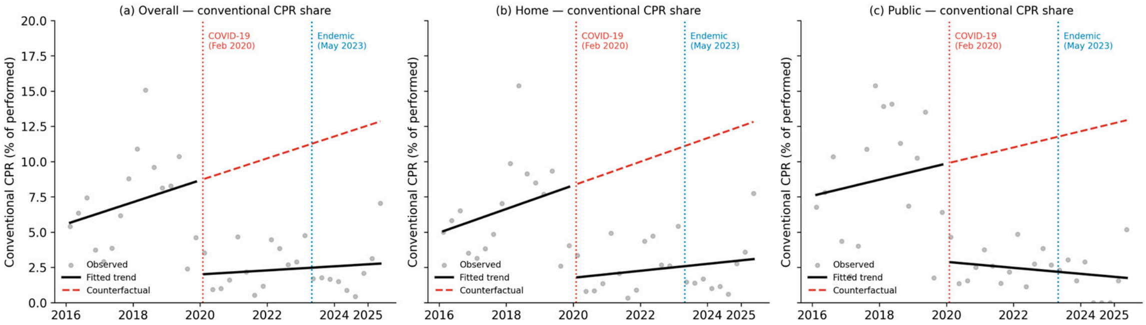

Figure S7. Mirror view: ITS of compression-only CPR share — abrupt level jump at COVID, sustained at >97% in all three strata.

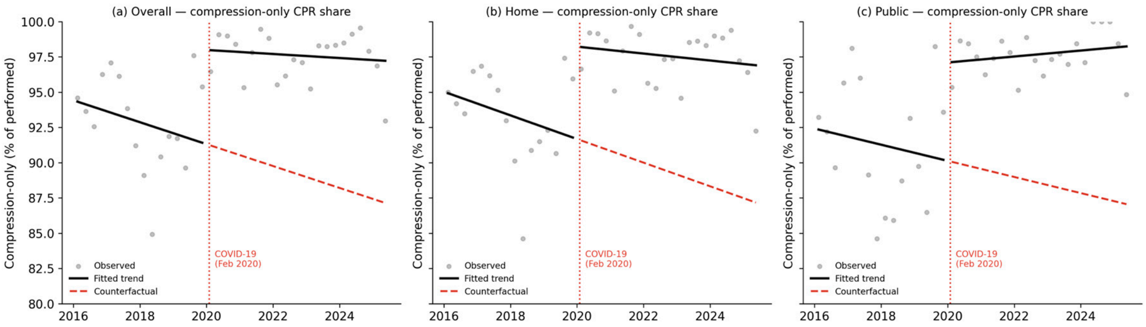

Figure S8. Three-level composition of the bystander response (no bystander CPR, compression-only, conventional) as a percentage of all OHCA with known bystander-CPR status: (a) annual trajectory 2016–2025; (b) by COVID-19 period. The abrupt fall in conventional CPR was mirrored by a rise in compression-only, whereas the proportion receiving no bystander CPR did not increase during the pandemic (45.5% → 41.9% → 43.8%), indicating method substitution rather than loss of responders (3 × 3 chi-square P<0.001).

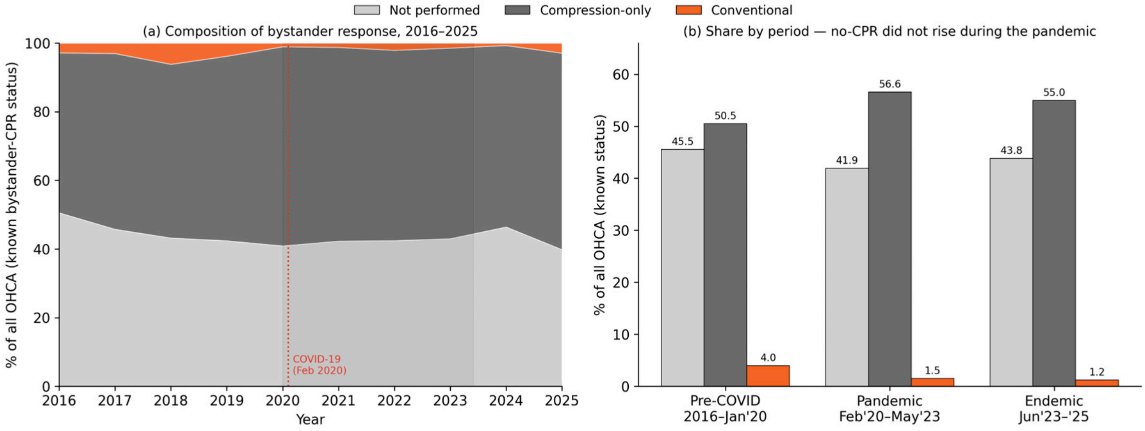

Supplement: Supplementary file 1 [file jcm-15-05469-s001.zip › jcm-4387019_Supplementary.pdf]
